# Cranio-Maxillo-Facial Reconstruction with Polyetheretherketone Patient-Specific Implants: Aesthetic and Functional Outcomes

**DOI:** 10.3390/jpm14080849

**Published:** 2024-08-10

**Authors:** Ylenia Gugliotta, Emanuele Zavattero, Guglielmo Ramieri, Claudia Borbon, Giovanni Gerbino

**Affiliations:** Maxillo—Facial Surgery Unit, Department of Surgical Sciences, AOU Città della Salute e della Scienza, University of Turin, Corso Bramante 88, 10126 Torino, Italy; emanuele.zavattero@gmail.com (E.Z.); guglielmo.ramieri@unito.it (G.R.); claudia.borbon@unito.it (C.B.); giovanni.gerbino@unito.it (G.G.)

**Keywords:** custom-made implant, patient-specific implant, maxillo-facial personalized reconstruction

## Abstract

Background: Reconstructing cranio-maxillo-facial defects presents significant challenges. This study evaluates the results of polyetheretherketone patient-specific implants (PEEK PSIs) in primary and secondary cranio-maxillo-facial reconstructions, with a focus on aesthetic and functional outcomes and long-term complications. Methods: From October 2009 to February 2023, 45 patients underwent cranio-maxillo-facial reconstructions with PSIs. Patients aged 18 years or older, with a minimum follow-up period of 12 months, were included. The morpho-functional outcome was evaluated through a modified Katsuragy Scale, the Visual Analogue Scale (VAS) for pain, and four FACE-Q|Aesthetics© scales. Results: In total, 44 PSIs were placed in 37 patients (51.3% males; mean age 45.1 years). The main cause of the defect was the resection of a tumor (55.4%). Mean follow-up was 78.6 months. Clinical evaluations showed an improvement in the postoperative period both in patient’s and surgeon’s scores (*p*: 0.01 and *p*: 0.002, respectively). Subgroup analysis confirmed a significant improvement in patients undergoing cranioplasty (*p* = 0.02) and mandible reconstruction (*p* = 0.03). No cases of prosthesis dislocation, rupture, or long-term infection were recorded. Conclusions: PEEK PSIs offer significant advantages in craniofacial reconstructions. Despite challenges in predicting soft tissue adaptation, overall patient satisfaction was high with no long-term complications. Future improvements should focus on predicting and enhancing soft tissue adaptations.

## 1. Introduction

Reconstructing defects in the cranio-maxillo-facial region poses a considerable challenge due to its complex anatomy characterized by different shapes, curvature planes, and soft tissue thicknesses [1].

Nevertheless, the postoperative re-establishment of the face contour is important for both aesthetic and functional reasons, enhancing the overall quality of life and social and psychological well-being of the patient. Therefore, it has become a surgical priority [2,3,4].

The use of various prefabricated patient-specific implants (PSIs) made from titanium, polyethylene, polyetheretherketone (PEEK), and other bioinert materials has been reported as a valuable procedure in the reconstruction of maxillo-facial defects [5,6,7,8,9,10,11].

In delayed reconstruction procedures, the use of digitally designed patient-specific alloplastic implants has been reported to reduce the time of surgery and described as an effective technique in craniofacial reconstruction [3,5,6,7,8]. In single-step resection and reconstruction procedures, the accuracy of the treatment plan transfer to the operating room depends on computed designed custom-made cutting and drilling guides together with the surgeon’s experience [6].

In this report, the authors describe a long-term experience with a significant follow-up in primary and secondary reconstruction of cranio-maxillo-facial defects with custom-made prefabricated PEEK PSIs, reporting the aesthetic and functional outcomes and long-term complications.

## 2. Materials and Methods

From October 2009 to February 2023, 45 patients underwent reconstruction of a cranio-maxillo-facial defect using a PSI at the Division of Maxillo-Facial Surgery and Division of Neurosurgery, AOU Città della Salute e della Scienza, University of Turin, Italy.

Patients were considered eligible for reconstruction with PEEK PSIs when they presented craniofacial defects or asymmetries not involving the dentoalveolar bone and requiring surgical reconstruction or correction that could not be easily achieved with less invasive soft tissue ancillary procedures. The inclusion criteria for the study were (1) craniofacial reconstruction with PEEK PSIs; (2) age over 17 years at the time of surgery; (3) minimum follow-up period of 12 months; and (4) availability of preoperative and postoperative clinical and medical records.

Patients undergoing exclusively post-traumatic orbital wall reconstruction and those not meeting the inclusion criteria were excluded from this study. 

The collected data included patient demographics, diagnoses, medical records, operative reports, imaging studies, reconstruction types, sites of reconstruction, utilization of cutting guides, postoperative complications (including the necessity of implant removal), and subsequent corrective procedures.

The clinical morpho-functional outcome was assessed through the surgeon’s visual evaluations and patient satisfaction surveys. Both patients and surgeons were presented with a modified Katsuragy Scale, a validated tool that evaluates the symmetry of the facial contour and the visibility of scars on the face both pre-surgery and post-surgery [12].

All patients were asked to complete the Visual Analogue Scale (VAS) of pain, a 0 to 10 scale (from “no pain” to “worst possible pain”), before surgery and 12 months after surgery. 

Finally, four FACE-Q | Aesthetics© scales (early life impact of treatment scale; satisfaction with outcome scale; psychological and social function scales) were administered to all patients. FACE-Q | Aesthetics© is patient-reported outcome (PRO) instrument comprising three modules; each module contains different rating scales that can be used independently. The Italian version of the selected scales and their respective score conversion tables have been provided by Memorial Sloan Kettering Cancer Center (New York, NY, USA) [13]. The presence of postoperative complications was monitored and recorded by the surgeon by a clinical examination during the hospital stay and at one-week, one-month, and three-month follow-up outpatient visits. 

### Surgical Planning

Preoperative high-resolution spiral CT datasets were acquired according to the guidelines provided by the manufacturer (Depuy Synthes^®^, Zuchwil, Switzerland and KLS Martin^®^, Tuttlingen, Germany). The CT data were saved as uncompressed DICOM files and sent to the manufacturer. In a web conference, the features, shapes, and characteristics of the implants were discussed and projected by the operating surgeon and manufacturer engineers to define the requirements for the prosthesis design. The plan was then revised by the company engineer and sent back to the surgeon for final approval.

In unilateral cases and when possible, a mirroring technique was used. In cases of bone resection and primary reconstruction, cutting guides were used for resection. In these cases, the shape of the cutting guide is planned together by the surgeon and the engineer, and the osteotomy is virtually simulated; then, the implant is designed according to the planned resection. 

## 3. Results

A total of 43 patients underwent reconstruction with patient-specific implants between October 2009 and February 2023. One patient passed away following a relapse of frontoparietal meningioma, while five others were lost to follow-up and thus could not be included in this study. A total of 37 patients were included (18 women and 19 men) with a mean age of 45.1 ± 13.5 years (range, 17–68 years). The mean follow-up duration was 78.6 months. In some patients, more than one implant was placed in the same procedure, resulting in a total of 44 PSIs being positioned. Table 1 summarizes the characteristics of the patients included in this study.

In the majority of patients (18 patients, 48,6%), the defect was caused by the resection of a tumor.

**Figure 1:**
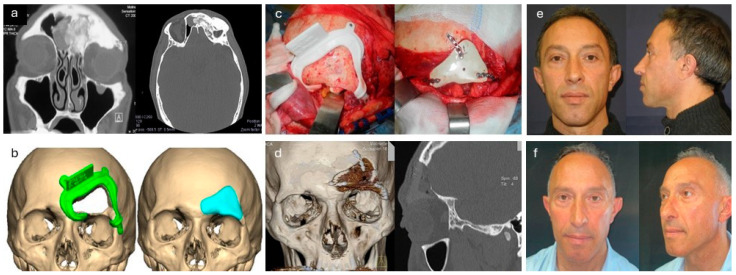
Resection and primary reconstruction of a fronto-orbital osteoma. (**a**) Preoperative CT scan revealing a heterogeneous fronto-orbital expansive process extending to the roof of the orbit and frontal sinus. (**b**) Customized cutting guide implant digital planning. (**c**) Intraoperative placement of the cutting guide for executing the osteotomy and subsequent fixation of the implant. (**d**) Postoperative CT scan. (**e**) Aesthetic outcome at the 3-month follow-up. (**f**) Aesthetic outcome at the 24-month follow-up.

**Figure 2:**
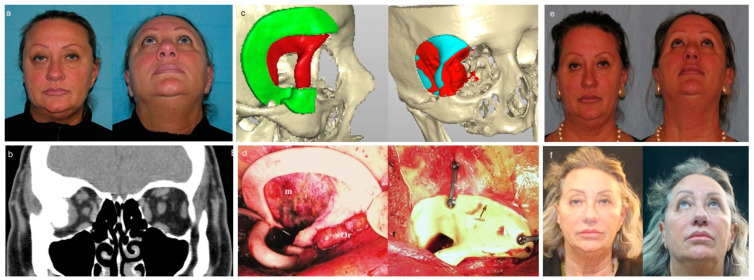
Resection and primary reconstruction of intraosseous meningioma. (**a**) Preoperative facial views of right exophthalmos and dystopia. (**b**) Preoperative coronal computed tomogram displays hyperostotic bone owing to intraosseous meningioma. (**c**) Three-dimensional model based on data from computed tomogram (green, cutting guide; red, custom-made implant, red and light blue, planned resection). (**d**) Intraoperative use of cutting guide to outline the area of resection that matches the size of the polyetheretherketone implant (m, meningioma; Or, right orbit) and reconstruction of bone defect with a polyetheretherketone patient-specific implant fixed in position with plate and screws. (**e**) Postoperative facial views show good functional globe position and excellent postoperative cosmetic appearance at 12-month follow-up. (**f**) Aesthetic outcome at the 36-month follow-up.

Ten patients (55.5%) had benign lesions that were removed simultaneously with the reconstructive procedure, except for one case of meningioma that was removed elsewhere and was reconstructed secondarily. In these patients, the excision of the tumor was carried out with the aid of custom-made cutting guides. (Figure 1 and Figure 2). 

The pathology reports confirmed six cases of meningioma, two of osteoma, and two of intraosseous low-flow arteriovenous malformation.

Eight patients had malignant conditions (five squamous cell carcinomas and three sarcomas); the reconstruction of these patients occurred secondarily, at least 5 years after the resection surgery.

The second most frequently reported cause of defect was facial trauma (38%), followed by congenital malformations (10.8%); in one case, the defect resulted from osteomyelitis subsequent to a decompressive craniotomy (2.7%). 

In cases of trauma, reconstruction was always carried out secondarily, following the improvement of the overall clinical conditions and the complete healing of fractures and soft tissues.

Out of the four patients with congenital malformations, two had previously undergone orthognathic surgery; hence, the reconstruction with a PSI was considered as secondary.

Overall, 12 patients underwent primary reconstruction, and 25 underwent secondary reconstruction.

**Figure 3:**
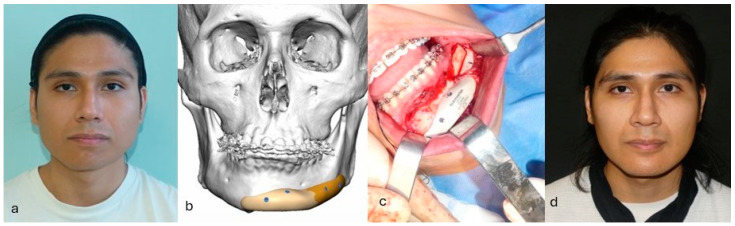
Correction of congenital mandibular asymmetry using two PEEK implants. (**a**) Patient with congenital asymmetry of the lower third of the face. (**b**) Planned correction of the asymmetry. Given the good occlusal compensation achieved through orthodontic therapy, a decision was made for a camouflage intervention using a patient-specific implant instead of opting for orthognathic surgery, which would have necessitated a new orthodontic treatment. (**c**) Intraoperative positioning of patient-specific implant. (**d**) Patient at 3-month follow-up after surgery.

Most of the implants were used for cranioplasty (54.5%); in eight of these cases, the implant extended to the orbital superior or lateral rim and/or walls. Of the remaining implants, eight were used for the reconstruction of the zygoma (including two extending to the lower orbital rim) and six were used for mandibular reconstruction (Figure 3); six were positioned within the temporal fossa to fill the volume loss resulting from the use of the temporal muscle flap following the removal of maxillary or orbital tumors. 

A total of 16 complications occurred in 13 patients (35.1%). In nine patients (24.3%), the postoperative course was complicated by a seroma, leading in two cases to wound dehiscence. In two patients, wound dehiscence occurred in the absence of a seroma. In three cases, an infection occurred, leading in two cases to the removal of the prosthesis (4.5% of 44 PSIs). The placement of multiple prostheses in the same surgical procedure was not found to be statistically associated with a higher risk of complications (single implant: 36.6% vs. multiple implants: 28.8%; *p*: 0.7). Despite the long follow-up period, no cases of prosthesis dislocation, rupture, or long-term infection were recorded.

In 10 cases, it was necessary to enhance the obtained result with additional surgical interventions. Six patients underwent lipofilling of the soft tissues above the implant, one patient underwent blepharoplasty, and one patient underwent both. In one case, scar revision was necessary. 

In 8 out of 10 of these patients, the additional surgical interventions on soft tissues were part of the initial therapeutic plan, which was expressly staged in two procedures. Therefore, these interventions cannot be considered unexpected. 

In seven cases (18.9%), the outcome of the intervention was deemed entirely satisfactory by both the patient and the surgeon, with no need for further surgical corrections. In 19 cases (51.3%), the patient considered the result satisfactory, but according to the surgeon, additional corrections could have been beneficial. In 11 cases (29.7%), both the patient and the surgeon recognized the need for secondary corrections. 

Regarding the clinical morpho-functional evaluation with the modified Katsuragy Scale, both in the scores given by patients and those given by the surgeon, there was an improvement in the postoperative period compared to the preoperative situation. Both of these variations were found to be statistically significant (*p*: 0.01 and *p*: 0.002, respectively) (Table 2). 

Pain did not appear to be a common symptom in the patients included in this study. After the intervention, a decrease in the average pain reported by the patient on the VAS scale was recorded, but this reduction did not prove to be statistically significant (2 vs. 1.3; *p*: 0.2) (Table 2).

Regarding the FACEQ assessment scales, 34 patients (91.9%) either fully agreed or partially agreed with the following statement: “I am pleased with the results of the intervention”. The remaining three patients included the two who had to have the prosthesis removed due to an infection. More than 80% of the patients either agreed or partially agreed with the statement “I like myself”, and 86.5% with the assertion “I am accepting of myself”. Regarding the social sphere, 86.5% of the patients felt they make a good first impression and were confident in new social situations. Regarding the impact on the quality of life during the postoperative period, no patient felt regret about the surgery or wondered if it was worthwhile (Table 2).

The analysis of morphological and functional outcomes in the subgroups divided based on the location of the reconstruction confirmed a statistically significant improvement in the morphological evaluation using the modified Katsuragy score in patients undergoing cranioplasty (*p*: 0.02) and mandible reconstruction (*p*: 0.03 and *p*: 0.02 by the patient and the surgeon, respectively).

## 4. Discussion

Reconstructing the craniofacial region poses challenges in both technical execution and aesthetic outcomes due to the complex anatomy, patient expectations, and defect uniqueness [2]. Despite these difficulties, reconstruction is strongly advocated to ensure biomechanical stability, protect the brain, preserve ocular function, and enhance cosmetic outcomes, contributing to the satisfaction of both the patient and the surgeon, as well as to the patient’s overall quality of life [14,15].

Determining the ideal material and technique for craniofacial reconstruction has been extensively discussed in various publications, resulting in numerous proposed solutions with acceptable outcomes.

Titanium is widely used in biomedical applications due to its high strength-to-weight ratio, fatigue and corrosion resistance, and biocompatibility. However, it has limitations, such as low thermal conductivity, a small modulus of elasticity, and difficulties with machining, which complicate the production of complex profiles and precise components [16].

High-density polyethylene solid implants are commonly used for augmentation and reconstruction, with the porous variety being particularly prevalent. Porous polyethylene (Medpor, Porex Surgical, Inc . Portage, Michigan, United States of America) is strong, nonbiodegradable, inert, and supports tissue ingrowth with minimal inflammation. Recently, ultrahigh-molecular-weight polyethylene (UHMWPE) implants that further enhance tissue integration have been developed. However, this porosity can complicate implant removal, potentially causing significant bleeding and damage to the surrounding soft tissues [6].

Polyetheretherketone (PEEK) is highly versatile and known for its excellent thermal, mechanical, and chemical properties, making it ideal for prosthetics and implants. Its low elastic modulus, akin to human bone, helps manage stress and tension, and it is biocompatible and adaptable to both conventional and CAD/CAM shaping methods. PEEK’s stress-absorbing and fracture-resistant qualities, along with its low reactivity, make it a strong alternative to other alloplastic materials [17]. 

Autologous bone grafts and free microvascular flaps can be considered good alternatives for the reconstruction of bone defects in the head and neck region, since they allow the use of autologous bone. However, bone grafts pose challenges for larger reconstructions due to insufficient donor material, limiting their use to small defects (less than 3 or 4 cm). Conversely, while free flaps increase the possibility of reconstructing large bone defects, they also significantly increase the complexity of the surgery and the risk of failure due to vascular issues. In both cases, there is the added risk of surgical complications related to the donor site, and shaping the harvested bone to reconstruct the complex anatomy of the facial bones can be extremely challenging. Additionally, the transferred bone may undergo remodeling and atrophy, which do not ensure long-term stability of the shape and volume of the reconstructed site [18,19,20].

The progress in additive manufacturing and CAD/CAM technologies has greatly enhanced the evaluation and surgical planning of craniofacial defects, and it has also opened new possibilities for creating customized alloplastic cranial prostheses [1,3,21]. 

Three-dimensional printing is an additive manufacturing (AM) technique used to create products with complex geometries and structures based on three-dimensional (3D) models. The most widely used 3D printing method is fused deposition modeling (FDM) due to its simplicity, adaptability, speed, cost-effectiveness, and minimal waste. The process consists of melting thermoplastic filament at a nozzle to create semi-liquid material, which is then deposited layer by layer to form 3D objects [22].

Patient-specific implants present the notable advantage of being customized to the shape and size of the defect prior to surgery. This customization not only reduces operative time but also minimizes the necessity for intraoperative modifications. Additionally, it enhances outcome predictability and avoids donor site morbidity [23,24].

When addressing benign lesions, primary reconstruction reduces the risk of suboptimal aesthetic and functional outcomes associated with a two-stage procedure. The primary challenge in these cases is to accurately define the shape and exact size of the defect to ensure a satisfying fit for the custom implant. Osteotomies are then executed with custom-made cutting guides in order to obtain the exact size, position, and shape of the defect. In accordance with Eppley et al. [19], the authors propose a cautious approach in estimating the amount of bone to be excised to avoid gaps between the implant and bone resection edges in cases of underestimated osseous infiltrations. 

The utilization of cutting guides not only facilitates easier and more predictable osteotomies but it also aids in the fixation of implants, minimizes surgical morbidity, and diminishes the intraoperative time [1].

The cutting guide should fit in a unique position, wrapping around key anatomical landmarks to avoid any misplacement. In certain cases, particularly involving the cranial vault where cutting guides are more prone to sliding, an intraoperative navigation system can be very helpful. STL files of CAD/CAM-generated cutting guides and patient-specific implants (PSIs) can be uploaded into the navigation system to verify the precise transfer of the surgical plan to the operating field. Other investigators have proposed different methods, such as navigation-guided craniotomy [25], and rubber [18] or aluminum [25] templates fixed to the skull with bones and screws.

In cases of secondary reconstruction, a specific implant is planned to reconstruct a pre-existing defect. This technique is reliable and predictable, but previous soft tissue reconstruction and integrity is a prerequisite for success. When soft tissue defects or extensive scars are present, merely mirroring the bony component is often inadequate, and the hypercorrection of the bone defect to compensate for deficiencies in the overlying tissues can improve outcomes. The degree of hypercorrection necessary is not currently dictated by any algorithm; rather, it relies on meticulous preoperative patient analysis and the surgeon’s expertise in implant design.

In this study, the majority of patients expressed satisfaction with the morpho-functional outcome, and, in most instances, both the patients and surgeons acknowledged the benefits of the surgery. However, while mirroring the contralateral side guarantees a reliable and predictable reconstruction of the bone segments, predicting the adaptation of the soft tissue on the implant and correcting soft tissue volume deficiencies can be challenging.

As shown in Table 2, patients in whom the implant was used to fill the volume previously occupied by the temporal muscle are on average less satisfied with the outcome and have a generally worse perception of their social and psychological sphere. Conversely, patients undergoing cranioplasty or reconstruction of the lower third are considered on average more satisfied, as demonstrated by the statistically significant postoperative improvement in the morphological evaluation with the Katsuragy Scale. 

The authors believe that unsatisfactory outcomes, in the absence of complications, frequently result from difficulties in predicting the adaptation of the soft tissue on the surfaces of implants. This is particularly notable when the implant is used to correct a soft tissue defect or to replace bone segments in areas where the soft tissue exhibits scarring or displays asymmetry compared to the contralateral side. In these cases, the authors believe that the patient must be informed from the beginning about the possible need for additional soft tissue correction procedures after the placement of the customized implant to achieve the best possible outcome. A further advancement in the design of these implants should focus on developing algorithms that predict and simulate the adaptation of soft tissues to the implant. This would allow for the early identification of any potential need for ancillary procedures, which could be performed during the same intervention, thus improving the patient’s aesthetic outcome without the need for additional subsequent surgeries.

The potential for postoperative infection is the major disadvantage of PEEK implants [20,26,27]. In this case series, three cases of infection were documented. In all these patients, the implants were inserted through intraoral approaches, indicating a higher risk of contamination associated with these procedures compared to transcutaneous approaches. In addition to good perioperative infection prevention practices, the risk of infection can be minimized by thoroughly evaluating the feasibility, risks, and benefits of transoral approaches. It is imperative to discuss the potential risk of infection and consequent prothesis removal with the patient during the preoperative consultation.

## 5. Conclusions

PEEK patient-specific implants present numerous advantages in craniofacial defect reconstruction. These implants accurately replicate the 3D structure of anatomically complex areas, typically necessitating no additional adjustments during placement. This not only diminishes intraoperative time and morbidity but also enhances the aesthetic outcome. This study demonstrates that digital planning and the design of customized implants, using contralateral bone mirroring and guided osteotomies, enable surgeons to precisely predict the site, size, and shape of both the defect and the implant. This approach facilitates the most accurate reconstruction possible, resulting in satisfying functional and aesthetic outcomes and enhancing the overall quality of life for the patient. The extensive follow-up of patients enrolled in this study confirms that PEEK implants exhibit reliability and durability for craniofacial defect reconstruction even after many years, with no cases of long-term complications reported within this patient cohort. Finally, this study shows that while CAD/CAM technology enables a highly accurate reconstruction of bony segments, in some cases predicting the adaptation of soft tissues over the implants proves challenging and may result in unsatisfactory outcomes. An additional step towards improving the outcomes of these patients should focus on developing algorithms that predict and simulate the adaptation of soft tissues to the implant and allow to incorporate areas of the implant with structures designed for soft tissue suspension, ensuring better coverage and adaptation of the implant.

## Figures and Tables

**Table 1:** Details of patients underwent reconstruction of the craniofacial area with PEEK PSIs.

Patient No.	Age (y)	Gender	Cause of Defects	Timing	Site of Defects	No. of Implants	Complications	Other Corrections
1	28	M	Trauma	II	Zygoma	1	No	No
2	55	M	Trauma	II	Frontal bone	1	No	No
3	31	F	Tumor ^§^	II	Zygoma + Orbit	1	Infection	Scar correction
4	50	M	Trauma	II	Frontal + Zygoma	2	No	Lipofilling, blepharoplasty
5	58	M	Tumor ^+^	I	Frontal+ Orbit	1	No	No
6	50	F	Tumor ^§^	II	Zygoma	1	Infection	No
7	57	F	Tumor ^†^	I	Frontal+ Orbit	1	No	No
8	63	M	Tumor ^§^	II	Temporal + Zygoma	2	Dehiscence	No
9	31	M	Congenital	II	Mandibular Angle	1	No	Lipofilling
10	26	M	Trauma	II	Temporal	1	No	Lipofilling
11	26	M	Trauma	II	Parietotemporal	1	No	Scar correction
12	59	F	Tumor ^µ^	I	Temporal + Orbit	1	No	No
13	35	F	Tumor ^§^	II	Temporal + Zygoma	2	Seroma, dehiscence	Lipofilling
14	58	F	Tumor ^†^	I	Frontal+ Orbit	1	No	No
15	32	F	Congenital	I	Cranium, frontal	1	No	No
16	17	M	Tumor ^¶^	II	Temporal + Orbit	2	No	No
17	68	M	Tumor ^µ^	I	Temporal + Orbit	1	No	No
18	63	F	Tumor ^µ^	II	Temporal	1	Dehiscence	No
19	19	F	Tumor ^¶^	II	Zygoma+ Mand. Angle	2	Seroma, infection	Lipofilling
20	58	F	Trauma	II	Frontotemporal	1	No	Lipofilling
21	66	F	Tumor ^µ^	I	Temporal + Orbit	1	No	No
22	45	M	Tumor ^+^	I	Frontal	1	No	No
23	61	F	Osteomyelitis	I	Frontotemporal	1	Seroma	No
24	68	F	Trauma	II	Frontotemporal	1	Seroma	No
25	52	F	Tumor ^µ^	I	Temporal + Orbit	1	No	No
26	53	M	Trauma	II	Temporal	1	No	No
27	41	M	Trauma	II	Temporal	1	No	No
28	49	F	Trauma	II	Frontal	1	Seroma	No
29	56	M	Trauma	II	Temporal	1	Seroma	No
30	28	M	Trauma	II	Temporal	1	Seroma	No
31	57	F	Tumor ^µ^	I	Temporal + Orbit	1	Seroma	No
32	23	F	Congenital	II	Mandible angle	1	No	No
33	51	M	Tumor ^§^	II	Temporal bilateral	2	No	No
34	29	F	Trauma	II	Mandible body	1	No	Lipofilling
35	45	M	Trauma	II	Frontal	1	No	Blepharoplasty
36	35	F	Tumor ^¶^	II	Temporal	1	No	No
37	25	M	Congenital	I	Mandible body	2	Seroma, dehiscence	No

^§^ squamous cell carcinoma; ^+^ osteoma; ^†^ intraosseous low-flow arteriovenous malformation; ^µ^ meningioma; ^¶^ sarcoma. BSSO: bilateral sagittal split osteotomy; PMMA: polymethylmethacrylate. For patients no. 6 and no. 19, implant removal was necessary due to infection.

**Table 2:** Functional and morphological evaluation.

Variable	Total n (%); N = 37	*p* Value *	Cranioplasty n (%); N = 24	*p* Value *	Zygoma n (%); N = 8	*p* Value *	Temporal n (%); N = 6	*p* Value *	Mandible n (%); N = 6	*p* Value *
Modified Katsuragy Scale—Patient		0.01		0.1		0.8		0.09		0.03
Preoperative										
Poor	12 (32.4)		9 (37.5)		3 (37.5)		0		1 (16.7)	
Sufficient	5 (13.5)		2 (8.3)		3 (37.5)		2 (33.3)		0	
Good	20 (54.1)		13 (54.2)		2 (25)		4 (66.7)		5 (83.3)	
Excellent	0		0		0		0		0	
Postoperative										
Poor	6 (16.2)		4 (16.7)		3 (37.5)		0		0	
Sufficient	5 (13.5)		2 (8.3)		2 (25)		2 (33.3)		1 (16.7)	
Good	17 (45.9)		14 (58.3)		3 (37.5)		1 (16.7)		1 (16.7)	
Excellent	9 (24.3)		4 (16.7)		0		3 50)		4 (66.6)	
Modified Katsuragy Scale—Surgeon		0.002		0.02		0.2		0.5		0.02
Preoperative										
Poor	19 (51.4)		11 (45.8)		7 (87.5)		2 (33.3)		3 (50)	
Sufficient	6 (16.2)		3 (12.5)		1 (12.5)		3 (50)		1 (16.7)	
Good	12 (32.4)		10 (41.7)		0		1 (16.7)		1 (16.7)	
Excellent	0		0		0		0		0	
Postoperative										
Poor	6 (16.2)		3 (12.5)		4 (50)		1 (16.7)		0	
Sufficient	9 (24.3)		5 (20.8)		3 (37.5)		2 (33.3)		2 (33.3)	
Good	15 (40.5)		12 (50)		1 (12.5)		1 16.7)		0	
Excellent	7 (18.9)		4 (16.7)		0		2 (33.3)		4 (66.7)	
	Mean ± DS	*p* Value **	Mean ± DS	*p* Value **	Mean ± DS	*p* Value **	Mean ± DS	*p* Value **	Mean ± DS	*p* Value **
Preoperative VAS score	2 ± 2.7	0.2	2.3 ± 3	0.5	1.1 ± 1.7	0.5	0.8 ± 1.4	0.9	1.5 ± 2	0.4
Postoperative VAS score	1.3 ± 1.7		1.7 ± 1.9		0.5 ± 0.9		0.7 ± 1.1		0.3 ± 0.5	
FACEQ™ Satisfaction with outcome	66.6 ± 18.2		68.9 ± 16.9		57 ± 23.5		58.3 ± 10.2		66.6 ± 13.4	
FACEQ™ Psychological function	62.9 ± 16.7		65.3 ± 13.4		57.3 ± 29		47.7 ± 12.1		65.6 ± 9.9	
FACEQ™ Social function	68.8 ± 19		75.7 ± 16.3		56.8 ± 25.3		48 ± 7.3		60 ± 4	
FACEQ™ Early life impact of treatment	71.7 ± 15.2		69 ± 12		70 ± 18		72 ± 17.3		87 ± 15.6	

* Chi-square test was conducted; ** T-test was conducted.

## Data Availability

The raw data supporting the conclusions of this article will be made available by the authors upon request.
